# Improved Optical Flow Estimation Method for Deepfake Videos

**DOI:** 10.3390/s22072500

**Published:** 2022-03-24

**Authors:** Ali Bou Nassif, Qassim Nasir, Manar Abu Talib, Omar Mohamed Gouda

**Affiliations:** 1 Department of Computer Engineering, University of Sharjah, Sharjah 27272, United Arab Emirates; u19104867@sharjah.ac.ae; 2 Department of Electrical Engineering, University of Sharjah, Sharjah 27272, United Arab Emirates; nasir@sharjah.ac.ae; 3 Department of Computer Science, University of Sharjah, Sharjah 27272, United Arab Emirates; mtalib@sharjah.ac.ae

**Keywords:** deepfake, optical flow, tensor processing units (TPU), GPU, convolutional neural networks (CNNs)

## Abstract

Creating deepfake multimedia, and especially deepfake videos, has become much easier these days due to the availability of deepfake tools and the virtually unlimited numbers of face images found online. Research and industry communities have dedicated time and resources to develop detection methods to expose these fake videos. Although these detection methods have been developed over the past few years, synthesis methods have also made progress, allowing for the production of deepfake videos that are harder and harder to differentiate from real videos. This research paper proposes an improved optical flow estimation-based method to detect and expose the discrepancies between video frames. Augmentation and modification are experimented upon to try to improve the system’s overall accuracy. Furthermore, the system is trained on graphics processing units (GPUs) and tensor processing units (TPUs) to explore the effects and benefits of each type of hardware in deepfake detection. TPUs were found to have shorter training times compared to GPUs. VGG-16 is the best performing model when used as a backbone for the system, as it achieved around 82.0% detection accuracy when trained on GPUs and 71.34% accuracy on TPUs.

## 1. Introduction

Deepfake multimedia (manipulated images, video and audio) have grown to become more and more of a threat to public opinion [1,2]. These fake multimedia are easily spread all over the world thanks to social media platforms that connect people with a click of a button [3]. Seeing a manipulated deepfake video of a public figure can alter a citizen’s opinions or political stance within seconds. The term deepfake refers to manipulated multimedia generated using artificial intelligence (AI)-based tools [4].

The most disruptive type of deepfake is a manipulated video in which a target person’s face is replaced by another face while keeping the target’s facial expression [5]. Although these generated videos can be very realistic and hard to detect, they are very easy to create. The availability of a wide variety of images and online videos has helped to provide enough data to create a huge number of fake videos. Anyone can generate these videos by combining the data available with free and open-source tools such as FaceApp [6]. Some positive applications of deepfake tools can be seen in movie productions, photography, and even video games [7]. However, deepfake technology has been infamously used for malicious purposes, such as creating fake news [8]. To address the problem of the malicious use of deepfake technology, the research and commercial communities have developed a number of methods to verify the integrity of multimedia files and to detect deepfake videos. Most of the methods attempt to detect deepfake videos by analyzing pixel values [9,10]. These methods rely on the visual artifacts created while placing the fake face on the target face. The artifacts are significant because they represent missing information that the deep neural network did not see in the training data (e.g., teeth), sometimes because it is hidden behind another object (e.g., hair strand). The deep network cannot estimate the information and therefore assigns it a lower quality in the deepfake version, or even creates holes in these parts [11].

However, the situation has changed considerably with recent developments in deep learning networks [12,13]. These artifacts are now less common than before and can no longer be seen in the new videos [14]. On the other hand, extracting other useful data from the video’s spatial and temporal information has proved to be effective. The estimated pattern of apparent motion over the length of time is called optical flow. This paper discusses how optical flow information can be exploited to detect anomalies in manipulated videos.

**Figure 1:**
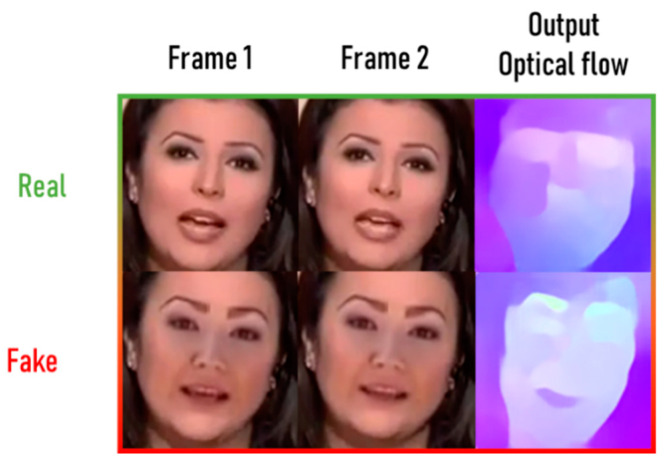
Difference between real (**top**) and fake (**bottom**) frames passed to optical flow estimator.

This research paper presents an effective technique to detect deepfake videos based on optical flow estimation. A sequence-based approach is used to detect the temporal discrepancies of a video. Inter-frame correlations are extracted using optical flow and are then used as input in a convolutional neural network (CNN) classifier. An example of applying optical flow estimation on real and fake frames can be seen in Figure 1. The proposed method is investigated using multiple neural networks to form the backbone of the system. TPUs are used to train another version of the system and a comparison is presented in this research. Furthermore, multiple methods of deepfake detection are tested on various datasets. Experiments are conducted using fine-tuning and augmentation techniques in order to improve the system. As this is the first work that uses TPU as training hardware to detect deepfakes, the research community can build and modify this work to explore TPU capabilities in detection methods. Furthermore, optical flow information in deepfakes has not yet been fully explored. This method can be utilized to build new detection methods.

The system achieves detection accuracy of 82% when trained on GPU. It has been trained and tested on multiple datasets. Multiple CNN models were tested to determine what should be used as a backbone for this system, and the VGG family had the best results. Augmentations were implemented to improve overall accuracy, but none of the augmentations were found to actually improve accuracy.

This paper has been laid out as follows: the related work on deepfake detection techniques is discussed in Section 2, while Section 3 presents background information related to the detection technique presented in this paper. Section 4 describes the methodology and modifications proposed in this paper to improve the overall accuracy of the system. Section 5 discusses the results where Section 6 presents the discussion. Finally, Section 7 draws conclusions.

## 2. Related Work

A brief overview of related research work is discussed in this section.

Matern et al. [11] proposed a method that focused on exploiting visual artifacts in generated and manipulated faces. The authors focused on the three most notable artifacts in the deployed detection method. The first artifact is the discoloration of the eyes. When a face generation algorithm creates a new face, the data points are interpolated between faces to find a plausible result. The algorithm tries to find two eyes from different faces that are matching in color. Utilizing the knowledge obtained by observing the fake data, the authors created their dataset from ProGan [15] and Glow [16] face generation datasets and generated deepfake and face2face [17] images using data from the Celeb-A dataset. Although the dataset used was limited to a small number, the results, as seen in Table 1, are very promising in this method.

Qi et al. [7] proposed an effective detection method utilizing remote visual photoplethysmography (PPG). Capturing and comparing the heartbeat rhythms of both the real and fake faces is the key idea of this method. The PPG monitors small changes of skin color caused by the blood moving through the face [18]. Using this information, PPG calculates an accurate estimation of the person’s heartbeat. The general concept assumes that fake faces should have a disrupted or non-existent heartbeat rhythm compared to the normal rhythms produced by real videos. The authors have done extensive testing on FaceForensics++ [9] and DFDC [19] datasets to demonstrate not only the effectiveness but also the generality of this method on different deepfake techniques.

Guera et al. [20] proposed a temporal method to detect deepfake videos. The system utilizes CNN to extract features from a video on the frame level. The extracted features are then used to train a recurrent neural network (RNN). The network learns to classify if the input video has been altered or not. The key advantage of this method is that it considers a sequence of frames when detecting deepfake videos. The authors chose to train the system on the HOHA dataset [21] because this dataset contains a realistic set of sequence samples from famous movies from which most deepfake videos are generated.

Amerini et al. [22] proposed a detection method exploiting the discrepancies in optical flow in fake faces as compared to real ones. The system transfers extracted cropped faces from video to PWC-Net [23], an optical flow CNN predictor. The authors conducted their tests on two well-known networks: VGG-16 [24] and ResNet50 [25]. Transfer learning was utilized to reduce training time and improve system accuracy. FaceForensics++ [9] uses multiple manipulation methods that the authors used in their tests. The three methods used are deepfakes, face2face, and face swap. Only the binary detection accuracy of face2face was shared in the research paper, with VGG-16 and ResNet50 detecting AI-generated faces with an accuracy of 81.61% and 75.46%, respectively.

Jeon et al. [10] proposed a light-weight robust fine-tuning neural network-based classifier capable of detecting fake faces. This system excels in its use of existing classification networks and its ease in fine-tuning these networks. The authors aim to reuse popular pre-trained models and fine-tune them with new images to increase detection accuracy. The system takes the cropped face images from the videos and transfers them to the backbone model, which is trained on a large number of images (78,000 images training/validation). The preliminary results show a substantial improvement in the accuracy of the models, with around 2 to 3% on the Xception [26] models and 33 to 40% for SqueezeNet models. The datasets used in this research paper included PGGAN [27], deepfakes, and face2face from the FaceForensics++ [9] dataset. The proposed augmentations and fine-tuning were applied only to the raw pixels of the image. However, discrepancies in the raw images, as mentioned before, are decreasing and may disappear entirely in the near future. Instead, implementing these techniques on the networks that analyze optical flow may increase the efficiency of these networks.

To overcome these limitations, we propose a system that is based on exploiting optical flow inconsistencies in videos to detect deepfake videos using pre-trained CNNs and augmentations. Table 1 shows a comparison between the various deepfake detection methods discussed in this section, including the method proposed in this work.

The main contribution of this work can be summarized as follows:

1. Improved accuracy: The proposed method achieves more accuracy overall than other the original method that utilized optical flow.
2. Detecting multiple deepfake techniques: Tests are conducted on several deepfake techniques including Deepfakes and face2face.
3. Experimenting with fine-tuning techniques: New augmentation techniques proposed by Jeon et al. [10] are implemented with the proposed method to try to improve accuracy in this paper.
4. Using TPU and GPU on the proposed method: The system is also trained on TPUs and compared with the GPU results as well.

## 3. Background

This section contains a brief overview of the inner mechanisms of deepfake and its types. Furthermore, different deepfake datasets are briefly discussed and compared based on dataset size and techniques used. An introduction about optical flow along with a short description of the most effective method used in this paper is also presented.

### 3.1. Deepfake

The power of AI has been harnessed to generate forged visual and audio content and new methods are continually being introduced. Most of these methods produce realistic video or audio segments that are difficult to recognize. The ability to produce such high-quality forensics is the result of advancement in generative adversarial networks (GANs) and autoencoders (AEs) [31].

GANs enhancements led to major improvements in image generation and video prediction. The fundamental principle of GANs is that a generator and a discriminator are trained concurrently. The generator produces fake reconstruction samples by using input from a random source [31]. The technique of the GAN is to make the generated reconstructions appear closer to a natural image. This is done by moving these reconstructions towards high probability regions in search space that contains photo-realistic images. The discriminator is trained to distinguish real samples of a dataset from forged reconstructions. The training of the discriminator ends when convergence occurs, which is done when the distribution produced by the generator matches the data distribution [9]. In more advanced approaches for deepfakes, GANs can be used along with Autoencoders (AEs) to generate fake images and videos.

Various methods are being used in deepfake generation techniques. They can be classified according to the type of media forged. Deepfake types can be classified as shown in Table 2.

### 3.2. Deepfake Datasets

As mentioned in the previous section, there are different categories of DeepFake manipulations. In this section, datasets related to face swapping and facial expression manipulation are presented. They are also used in this work as shown in Table 3.

FaceForensics++ [9] is one of the most utilized datasets in deepfakes. This dataset is generated from 1000 pristine videos available on Internet. Many manipulation techniques have been applied to generate fake videos from these 1000 videos. The techniques used can be classified into two categories: computer graphics-based and two-learning based approaches. Computer graphics-based approaches include such techniques as Face2Face and FaceSwap. Examples of the two-learning based approaches include DeepFakes and NeuralTextures. One of the features of the dataset is that it supports a variety of video qualities, which is an important factor in video forensics and the deepfakes paradigm.

Celeb-DF [14] is a large-scale video dataset that consists of 2 million frames that correspond to 5693 deepfake videos. This dataset is characterized by diversity in the gender, age, and ethnic group of its subjects, as its videos are sourced from Youtube. Using an enhanced deepfake synthesis method, fake videos are generated from the source videos. The recently introduced Celeb-DF v2 overcomes the shortcoming of the original version of the dataset, as it has significantly fewer notable visual artifacts.

A new challenging dataset that has been introduced lately is Deepfake Detection Challenge dataset (DFDC) [19]. This dataset consists of more than 100,000 videos and takes into consideration variability in gender, skin tone, age, varying lighting conditions, and head poses. Currently, the DFDC dataset is the largest publicly available face swap video dataset. The forgery videos are generated using several techniques including deepfake, GAN-based and non-learned methods.

### 3.3. Optical Flow

One of the key problems in computer vision is optical flow estimation. However, this field is making steady progress, which can be seen in the current methods on the Middlebury optical flow benchmark [35]. Optical flow estimation is the estimation of the displacement between two images and is conducted at the pixel level. Multiple approaches were introduced to make this type of estimation conclusive. Horn and Schunck introduced the variational approach, in which brightness constancy and spatial smoothness are coupled and passed to an energy function [36]. However, using energy functions is computationally expensive, especially for real-time applications. To overcome this problem, CNNs are adopted to maximize speed and minimize cost. One of the top-performing methods that use CNNs is PWC-Net [23].

**Figure 2:**
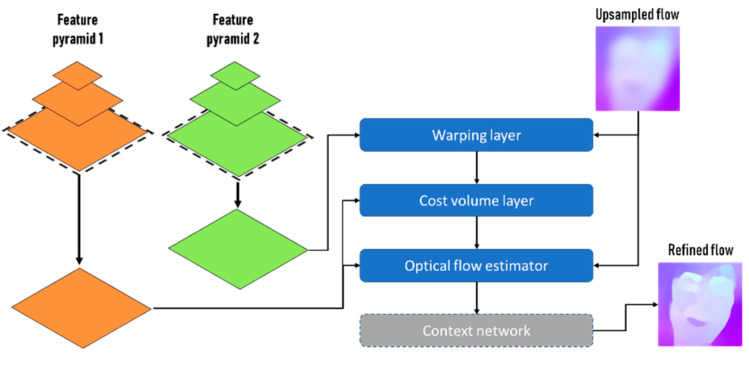
PWC-Net Network Architecture.

PWC-Net, as shown in Figure 2, utilizes pyramid, wrapping, and cost volume processing along with CNN, building a feature pyramid from the two input images. Unlike approaches such as FlowNet [37], which uses fixed-image pyramids, PWC-Net employs learnable feature pyramids [23]. A cost volume is constructed at the top level of the pyramid by comparing the features of a pixel in the first image with the corresponding features in the second image. The cost volume is constructed using a small search range, as the topmost levels have a small spatial resolution. The cost volume and features of the first image are passed to a CNN to estimate flow at the top level. PWC-Net passes to the next level the estimated flow that has been upsampled and rescaled [23].

PWC-Net warps feature the second image toward the first using the upsampled flow at the second level from the top. Using the features of the first image and the warped features, PWC-Net constructs a cost volume. This process repeats until the desired level is reached [23].

## 4. Materials and Methods

**Figure 3:**
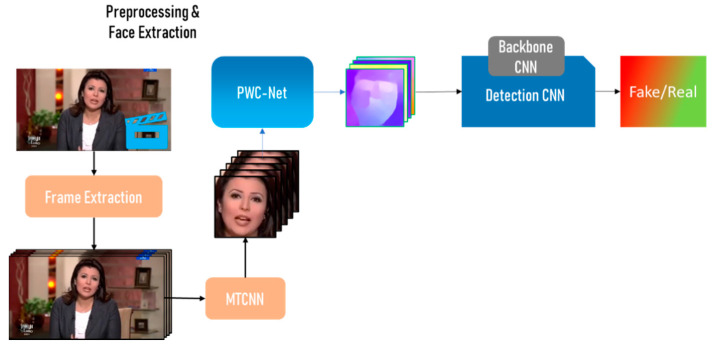
Overall proposed system architecture.

In this section, the proposed method is described. The method’s overall architecture is presented in Figure 3. The architecture uses several different approaches to improve the system’s overall accuracy. GPU-based, TPU-based, and augmented approaches of the proposed system are presented.

### 4.1. Proposed Architecture

As presented in Figure 3, the proposed system starts with a preprocessing stage in which the person’s face is extracted before analyzing the video. In this preprocessing stage, the frames of the video are extracted and saved on the system disk. The extracted frames are much larger in size than the source video. Furthermore, in this system, the interest region is the face. The frames are therefore passed to MTCNN [38], which detects the faces in the frames, and then to OpenCV [39], which crops the frames to contain only faces and ensures that the frames are all a fixed size.

The cropped frames are then passed to PWC-Net [23], which will process the frames in chronological order and extract the movement between the scene and the observer, which is done between two consecutive frames f(t) and f(t+1). The former process is called Optical flow. The information generated from this process is in a vector field format. Each value has a magnitude, which refers to the amount of motion in that pixel or point, and the direction of the scene’s motion between these two frames.

Each optical flow vector is visualized and presented as an RGB image. These frames are called optical flow frames or images. The pixel color represents the angle between the flow vector and the horizontal axis, while color saturation represents the intensity of the motion in this pixel. This step was taken in order to save training time and reuse the information obtained by the existing implementations and pre-trained networks trained on raw RGB images.

The optical flow images are then passed to Detection CNN, which uses a pre-trained CNN as a backbone. Transfer learning is adopted for this approach, as these networks are already trained on RGB images. Exploiting the knowledge they retain, additional training is conducted with three dense layers to fine-tune the network on the optical flow images.

The hypothesis for this architecture is that the optical flow should detect and show any discrepancies in motion that were added or synthesized into the frame. These differences should be noticeable when compared to the real parts of the frames that were created by the camera. The regions of interest are the eyes, the mouth, and the face outline. These regions are most likely to contain the said discrepancies, as deepfake synthesis algorithms struggle the most with these regions.

The results of the Detection CNN are evaluated using the test dataset, which is a also taken from the same dataset used in training. This dataset was not used in the training phase of the model. Algorithm 1 summarizes the algorithm used in this research work.
**Algorithm 1** Deepfake Detection Using Optical Flow ModelFor video V in the dataset
Extract frames from video V.Pass the frames to MTCNN.
Detect the face in video.Crop the face.Export the faces as images.Pass the frames sequences PWC-NetExport the optical flow sequence as RGB images.Prepare the model
Load the model trained on imagnet dataset.Remove the last classification layers.Freeze the model and keep the last three layers trainable.Add dropout layer with rate of 0.2Add dense layer with softmax.Train the model
Add reduce learning rate callback.Add early stopping callback.Start training the model.Evaluate the model

### 4.2. GPU Approach

**Figure 4:**
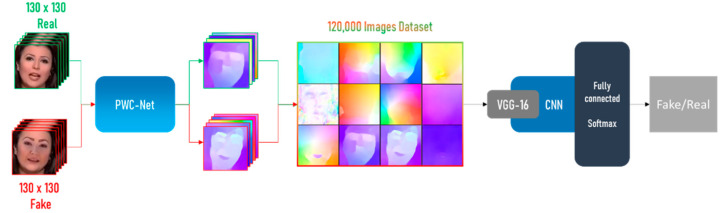
GPU architecture of the system.

The basic structure of the GPU approach is shown in Figure 4. The overall architecture of this approach replaces the Detection CNN with a CNN that was pre-trained using a GPU. Multiple networks have been tested, including but not limited to VGG-16 [24] and Xception [26].

The network uses the original top layer of the network. For example, VGG-16 uses 224 × 224 image size as input. Then, using the Keras [40] function Image generator, the images are scaled to the correct size for the network. As Keras retains all the weights taken from Imagenet [41], only the last three layers plus the dense ones are kept trainable. All other layers become untrainable. Finally, the last 2 layers are a dropout layer with rate of 0.2 and the dense layer is switched with a softmax dense layer that categorizes the frames as fake or real.

### 4.3. Augmentations and Fine-Tuning Approach

Jeon et al. [10] proposed a new self-attention module for image classifications called Fine-Tune Transformer. It was used with MobileNet Block to improve existing networks that were trained on deepfake images. Our methods of implementing this fine-tuning method on the proposed design is explained in this section, along with multiple augmentations.

**Figure 5:**
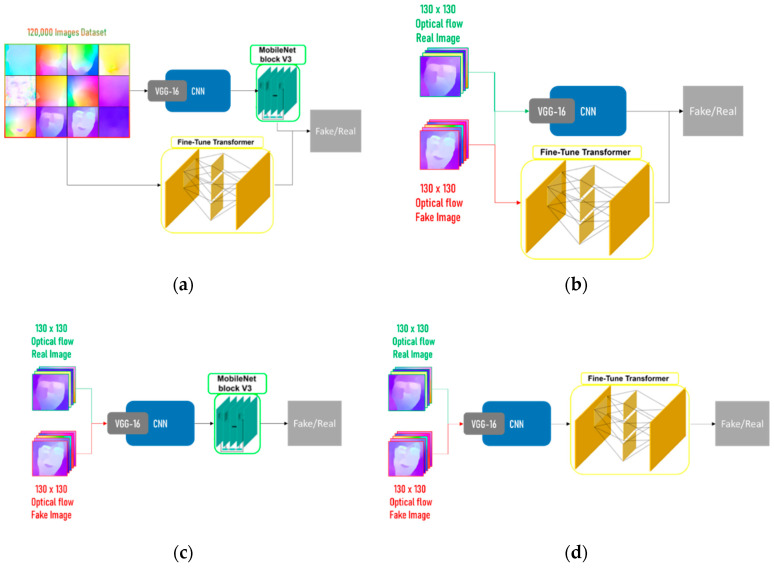
Four different augmentations: (**a**) Augmentation 1: applying the proposed augmentation by Jeon et al.; (**b**) Augmentation 2: training the FTT block alongside the CNN; (**c**) Augmentation 3: attaching MobileNet block at the end of the CNN; (**d**) Augmentation 4: attaching FTT block at the end of the CNN.

In order to apply the fine-tuning method as it was mentioned in the paper and as shown in Figure 5a, we take the pre-trained GPU approach CNN as the backbone and pass it to the MobileNet block. At the same time, the Fine-Tune Transformer (FTT) block is trained on the same dataset as the MobileNet. All other parameters are maintained exactly as in the GPU approach. Another augmentation as shown in Figure 5b is to train the FTT alongside the CNN, as this may increase the accuracy of the entire system. Augmentations 3 and 4 are very similar, placing different blocks after the CNN. MobileNet block is used in Augmentation 3 and FTT in Augmentation 4 as shown in Figure 5c,d, respectively.

### 4.4. TPU Approach

The Tensor Processing Unit (TPU) is a custom ASIC-based accelerator. It has been deployed in data centers since 2015, but access for academic purposes was only given in 2018. In most neural networks, TPUs speed up the inference stage, which is a crucial stage in which models are used to infer or predict the testing sample.

The brain of the TPU has a 65,536 8-bit Multiplier Accumulator (MAC) matrix unit with throughput that peaks at 92 TeraOps/second (TOPS) and with on-chip memory that is software-managed at 28 megabytes [42]. These specifications allow TPUs to handle a large number of data that GPUs cannot handle and still be extremely fast. Nevertheless, TPUs require extra effort from the programmers to create a working model that can run seamlessly on these units. Moreover, the TPUs utilize their own bfloat16 floating-point format, which supports only an 8-bit precision compared to the GPUs’ 24-bit of the 32-bit binary format. This may cause lower accuracies in some cases, which will be seen in the results section.

**Figure 6:**
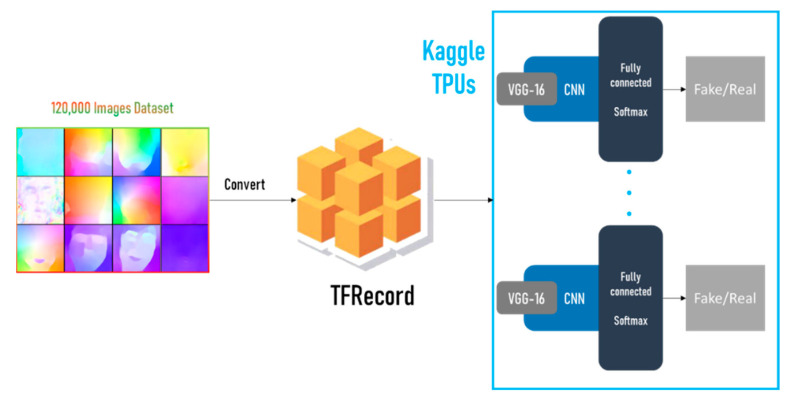
Basic architecture for the TPU approach.

Kaggle platform offers the latest TPU v3 for academic purposes. The proposed model is adjusted, as seen in Figure 6, to work on TPUs seamlessly. TPUs require image data to be in TFRecord format, which converts the images into binary strings along with their label. In this case, the image size is fixed, and a custom data generator is used to read the binary sequence and convert it back into an image. The data is distributed over 8 TPUs and the result is combined to enhance accuracy.

### 4.5. Extraction and Filtering

For each dataset used in this paper, frames of each video were extracted with python code using OpenCV [39]. The frames were then cropped by MTCNN [38] so that each image was of a fixed size and contained only the subject’s face, before transferring the images to the optical flow estimator. The images are first filtered manually, as the sequence of frames is very important in the optical flow extraction step.

Sorting by size is a simple method used to find any images that do not belong in a given sequence. Images with similar pixel values and density are more likely to have similar size and are therefore grouped together. Any foreign or unwanted frames are also grouped together, as they have different characteristics. If the unwanted frames do not affect the sequence, the frames are deleted. However, if they do affect the sequence, the video is either re-run through the MTCNN with a different threshold or removed entirely.

After the filter is applied, the frames are passed into a PWC-Net [23] optical flow estimator. This estimator works only on two frames at a time, given that is it provided two text lists containing the frames to be used. Therefore, the image fetch code used in FlowNet2 [43] is used to automate the process by providing the folder that contains the images. The number of frames transferred to the estimator should be double the number used for the dataset. The estimator extracts data from two consecutive frames, thereby cutting the number of produced images in half.

As mentioned before, for the TPU approach, the images should be in TFRecord format. The images were converted to a binary string and the label of the image into int32. Afterwards, the data are put in a list and divided into batches of 2071 per TFRecord.

### 4.6. Training Stage

The following tables show the training settings used in tests and experimentations. Table 4 shows the number of frames used, which is 120,000 optical flow images divided into 80-20-20 ratios. Further down, Table 5 shows the parameters used for each model in detail. In the training stage, the model utilized two callback functions the early stopping and reduce learning rate. The early stopping was employed to prevent overfitting on the training data and to reduce the training time if no improvement was seen in ten epochs. Moreover, the validation loss was monitored for every five epochs by the reduced learning rate callback. However, the learning rate was reduced only if the validation loss is deteriorating or stays the same over these five epochs.

## 5. Results

In this section, the results of all experiments conducted in this research paper are shown and evaluated. Furthermore, the results are compared with Amerini’s work [22], which is referred to in this section with “original” or “binary”. The experiments were done on an Ubuntu 18.04 LTS server PC with 64 GB RAM and RTX 2080 GPU with Intel^®^ Xeon(R) Silver 4208 CPU @ 2.10 GHz CPU. Kaggle platform provided the TPUs with 8 cores that were used for the TPU experiments in this section.

### 5.1. GPU Results

**Figure 7:**
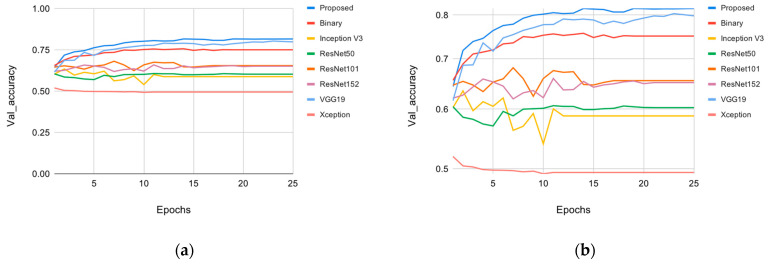
Backbone CNNs validation accuracy vs. epochs: (**a**) linear scale; (**b**) logarithmic scale.

The overall accuracy of the method proposed by this research paper, which was trained on the FaceForensics++ [9] dataset, is shown in Figure 7, with more details in Table 5. Figure 7 shows the validation accuracy results of multiple models trained on GPU. The various models can be placed into three categories: VGG, ResNet, and Inception. The VGG category included the top-performing models in terms of accuracy, and VGG-16 had the highest accuracy overall. VGG-19 was the second-best performing model, while the binary method, which uses VGG-16, performed third. The ResNet category included the ResNet family, with ResNet101 performing best in this category. The performance of ResNet152 was similar to that of ResNet101, but it ultimately had lower detection accuracy. ResNet50 performed the worst in this category. The Inception family included Inception V2 and the new model based on the Inception model, Xception. Xception performed the worst in this category and overall.

As seen in Table 6, when Xception was used as a backbone for this system, it performed the worst, with accuracy measured at 52%. ResNet50 performed slightly better than the Xception model with 60.64% accuracy. The other members of the ResNet family, ResNet101 and ResNet152, performed better with 65.9% and 65.8% accuracy rates, respectively. Inception did not perform well, with an accuracy of 62.1%. The common factor among all the previously mentioned models is that all of them use a depth-wise separator convolution layer in their model. This layer’s counterpart, the depth-wise convolution layer, is used in the VGG family. The original method proposed by Amerini [22] used binary classification and achieved 75.27% accuracy in detecting deepfake images from the FF++ dataset. The second-best performance was achieved by the VGG-19 model, with 80.1% accuracy. VGG-16 performed the best, with 82.0% accuracy, and was the third-fastest model out of all tested models.

**Figure 8:**
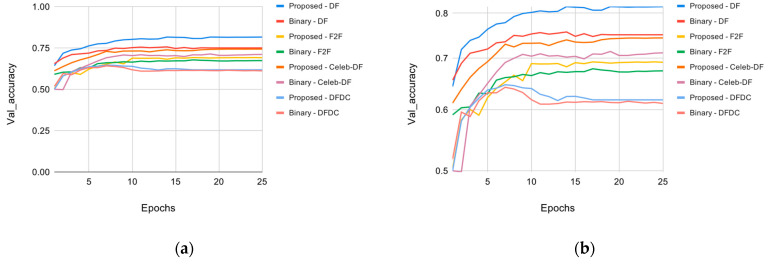
Accuracy comparison between the proposed and the original trained on different datasets: (**a**) linear scale; (**b**) logarithmic scale.

Figure 8 shows the accuracy results of the best performing model, which is VGG-16, trained on different datasets and compared with the original method trained on the same datasets. Table 7 presents more details of the same test.

The proposed model attained much higher accuracy than the original method with minimal modifications. The original method performed binary classification and used a fully connected sigmoid output layer. The simple modification of using the categorical classification and softmax output layer improved its accuracy. Another modification in the model was the input image size. The original work used 300 × 300 images that were scaled down to 224 × 224. Using 130 × 130 images and scaling them up to 224 × 224 also helped increase the accuracy of the model. 

Table 8 shows the cross-validated results of the best-performing model, which is the VGG-16 model, on four different datasets. The datasets include FaceForensics++ Deepfake and Face2Face, DFDC, and Celeb-DF. The results are shared in area under the receiver operating curve (AUROC). The curve shows the trade-off between the false positive rate (FPR) and the true positive rate (TPR) across different decision thresholds. The table shows that each model best detects the dataset that was used in training. Overall, the model trained on FaceForensics++ Deepfake dataset performed the best, at around 66.78% accuracy.

**Figure 9:**
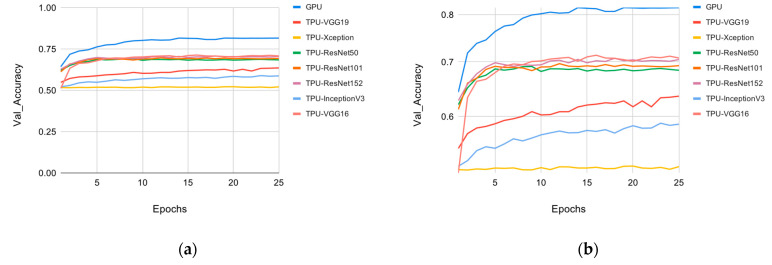
Comparison between the best performing GPU and other models trained on the TPU: (**a**) linear scale; (**b**) logarithmic scale.

Figure 9 shows the comparison between the best performing GPU and other models trained on the TPU. Similar to the GPU training results, three clear categories for the models can be seen in the graph. The only model that deviated from its category was VGG-19, which performed much worse than VGG-16. The accuracy of the VGG-16 GPU exceeded the same model’s TPU accuracy by 12%. However, the speed with which the training was completed on TPU was phenomenal: around eight times faster than the GPU training periods, as shown in Table 9 in the next section.

### 5.2. TPU Results

As stated above, training was completed on TPU around eight times faster than on GPU. Furthermore, there was a noticeable increase in the accuracy of the models to accompany the decrease in training time. ResNet152 recorded one of the worst training times among all GPU-trained models, with 1994 s per epoch and 839 min for all 25 epochs, with an accuracy of 65.79%. Comparing these values with the ResNet152 TPU results, there is a major improvement in training time, with 110 s per epoch and a total time of 46.3 min, but also in accuracy, with 70.50% accuracy. Table 9 demonstrates the different CNN models trained on TPU compared with the best-performing GPU model.

## 6. Discussion

In this section, the augmentation experiments in this research work are evaluated and discussed. Furthermore, the limitations and future work of this research paper are discussed afterward.

### 6.1. Augmentation Experiments

**Figure 10:**
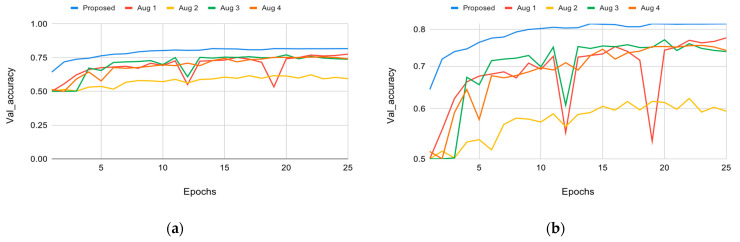
The effect of each Augmentation on validation accuracy over 25 epochs: (**a**) linear scale; (**b**) logarithmic scale.

After implementing the four different augmentations proposed in the methodology section, a comparison is done in this section. As shown in Figure 10, without augmentations, the model proposed by this paper had the highest accuracy of all the models compared. All augmentations made to the model performed worse than the model with no augmentations.

As shown in Table 10, the accuracy dropped significantly by around 25% with Augmentation 2. Accuracy was measured at 61.5%, and training was done in parallel with the FTT block. The augmentation not only decreased the accuracy, but also increased training time by approximately three times more than that of the standard model with no augmentations. Using the FTT block alone after the backbone CNN did not provide any useful information; in fact, it decreased the system’s accuracy by around 8% in Augmentation 4. Furthermore, the results of Augmentations 1 and 3 showed decreased accuracy by 6% and 8%, respectively. These tests allow us to conclude that the proposed design without augmentation excels over the augmented models, with accuracy measured at 82% and with the shortest training time as well. The causes of the degradation in the accuracy of the augmented models are discussed in the limitations section.

### 6.2. Limitations and Future Work

Depth-wise separable convolution reduces the number of parameters while achieving the same results as depth-wise convolution. It also produces new features after each convolution, combining image channels to create new features, which depth-wise convolution does not do [45].

Extracting more features from the optical flow image can make the model under-fit the results because the extra details are not necessary and can be detrimental to the process. This can be observed in both the augmentations and other CNN results. In the applied augmentation, MobileNet [45] and the FTT [10] both utilize depth-wise separable layers. Furthermore, Xception [26], Inception [44], and the ResNet [25] family also rely on the same layer in their model. This explains why the VGG [24] family had the highest accuracy when it was trained on images extracted from optical flow information.

Another major limitation of this method is the fact that the sequence or the flow of the frames must be preserved. Any false faces or unrelated images placed in the sequence will result in a false optical flow image.

Exploring other color spaces can improve the accuracy of the models and the processing time of the images. The HSV space has the important feature of decoupling the intensity and hue components, which can be useful in processing the resulting optical flow images [46].

Passing different resolutions of face images as input can affect the performance of the system as seen in this research work. Therefore, experimenting with different resolutions may improve the accuracy of the model.

As seen in the results above, TPUs improve the time required to train and evaluate models. For future work, training on TPUs with proper parameters and enough effort can lead to a huge improvement in deepfake detection. As seen in the results, the training time is almost four times faster than using the GPU. Moreover, in some instances as the ResNet, it performed better than the GPU with the same input image size of 224 × 224. It will be necessary to explore more CNN models in the future to find the advantages and limitations of this approach, similar to what was done in examining the depth-wise separable layer.

## 7. Conclusions

In this research work, optical flow was used to detect deepfake videos by detecting inconsistencies in video frames. The frames were cropped and transferred to a PWC-Net optical flow estimator, which is a state-of-the-art tool created by NVIDIA. The resulting frames were then sent to pre-trained image classification models such as VGG-16 and Xception in order to reuse the training weights to cut down training time. These models were trained on GPU. The best performing model was VGG-16, with 82.0% detection accuracy. Furthermore, the models were trained on TPUs. TPU training results proved that there is great potential for using this hardware in deepfake detection and classification methods. The training time was cut by almost eight times for the same model trained on a GPU. Moreover, some models improved detection accuracy compared to the GPU results. In addition, four different augmentations were applied to the proposed method in order to improve accuracy. However, detection accuracy was negatively affected by these augmentations. Most of the tested models used depth-wise separator layers, which performed poorly in this method. The top-performing model relied on the depth-wise convolution layer. The augmentations also used depth-wise separator convolution layers, which affected the accuracy of the VGG-16 model negatively. Researchers can use this work as the starting point in TPU utilization in detecting deepfakes. Furthermore, it can be also used to explore the impact of augmentations on several existing CNNs. For future work, training on TPUs with proper parameters can significantly improve deepfake detection. As the results showed, the time taken in training is almost four times faster than using the GPU. Moreover, in some instances as the ResNet, it performed better than the GPU with the same input image size of 224 × 224. Furthermore, it will be necessary to explore more CNN models in the future to find the advantages and limitations of this approach, similar to what was done in examining the depth-wise separable layer.

## Figures and Tables

**Table 1:** Summary of related work in deepfake detection.

Research Paper	Year	Method	Domain	Datasets	Hardware	Accuracy
DeepRhythm [7]	2020	Heartbeat rhythms using PPG with attention network	Dual-spatial-temporal	FF++ * DFDC	GPU	Accuracy: 98.0%
FDFtNet [10]	2020	Augmentation of pretrained CNN	Pixel-Level detection	PGGAN, FF++ –Deepfake, FF++ –Face2Face	GPU	AUROC: 0.994 Accuracy: 97.02%
Face X-Ray [28]	2020	Detection of blending boundaries in the image	Pixel-Level detection	FF++ * DFDC DFD Celeb-DF	GPU	AUC: 95.4
Visual Artifacts [11]	2019	Visual artifacts (eyes, teeth and nose and face border)	Pixel-Level detection	Glow, ProGan, celeb-A	GPU	AUROC: 0.866
Optical Flow [22]	2019	Inter-frame correlations using optical flow	Spatio-temporal	FF++ –Face2Face	GPU	Accuracy: 81.61%
Recurrent Neural Networks [20]	2019	Recurrent Neural Network	Spatio-temporal	HOHA	GPU	Accuracy: 97.1%
FF++ –Xception [9]	2019	CNN-based Image classification	Pixel-Level detection	FF++ *	GPU	Accuracy: 96.36%
Eye Blinking [29]	2018	Discrepancies in eye blinking across the frames	Spatio-temporal	CEW	GPU	AUROC: 0.98
Edges & Optical flow [30]	2020	Edges of optical flow images with XceptionNet	Spatio-temporal	FF++ * DFDC-mini	GPU	Accuracy on DFDC-mini: 97.94%
Optical flow based CNN [2]	2021	Optical flow-based CNN	Spatio-temporal	FF++ *	GPU	Accuracy on Optical flow only: 82.99%
This research paper	2022	Inter-frame correlations using optical flow	Spatio-temporal	FF++ –Deepfake, FF++ –Face2Face Celeb-DF, DFDC	GPU, TPU	AUROC: 0.879 Accuracy: 82%

* Means all types of manipulation methods were used in the paper.

**Table 2:** Types of deepfake manipulation.

Type	Photo	Audio	Video
Description	This type includes manipulations done on images, i.e., to generate a non-existent face image.	This type includes any type of manipulation done on audio records, i.e., impersonating or changing a person’s voice.	This type includes manipulations done on videos.
Class	Face and body swapping.	Impersonating person’s voice. Changing a person’s voice. Speech to text usage to change part of audio to a specific text.	Face-swapping. Face-morphing. Full body puppetry.
Example	FaceApp [6].	Synthesizing Obama: Learning lip sync from audio [32]. Waveglow [33].	Face2Face [17]. Pose transfer [34].

**Table 3:** Deepfake datasets.

Dataset	Year	Size (Videos)	Techniques
FF++ [9]	2019	1000 real 7000 fake (all techniques)	Deepfakes, Face2Face, face swap, NeuralTextures
Celeb-DF v2 [14]	2020	590/5639	Deepfakes
DFDC [19]	2020	19,154/100,000	8 different deepfakes techniques

**Table 4:** Frames used in the experiments.

Dataset	Videos Used	Original Frames	Optical Flow Frames	Training/Validation/Test
FaceForensics++ –DF	631	240,000	120,000	80,000/20,000/20,000
FaceForensics++ –F2F	545	240,000	120,000	80,000/20,000/20,000
Celeb-DF	1254	240,000	120,000	80,000/20,000/20,000
DFDC	962	240,000	120,000	80,000/20,000/20,000

**Table 5:** Training parameters.

Approach	Optimizer	Learning Rate	Compiler Loss	Last Dense	Epochs
GPU	Adam	1e-4	categorical_crossentropy	2, softmax	25
GPU-Orignal	Adam	1e-4	binary_crossentropy	1, sigmoid	25
Augmented	Adam	Default	categorical_crossentropy	2, softmax	25
TPU	Adamax	1e-4	sparse_categorical_crossentropy	2, softmax	25

**Table 6:** Backbone CNNs accuracy comparison. Values in bold are the best values in each category.

Model	Time Per Epoch	Total Time	Accuracy
Inception V3 [44]	800 s	335 min	62.1%
ResNet 50 [25]	**77 s**	**33 min**	60.64%
ResNet 101 [25]	1207 s	507 min	65.89%
ResNet 152 [25]	1994 s	839 min	65.79%
Xception [26]	633 s	264 min	52.0%
VGG-19	698 s	294 min	80.1%
VGG-16 Binary (Amirini’s) [22]	446 s	187 min	75.27%
VGG-16 (Proposed)	440 s	183 min	**82.0%**

**Table 7:** Dataset evaluation on proposed vs. original. The highlighted values in bold are the best performing for each dataset.

Model	Dataset	Accuracy	Overall Accuracy
Proposed	FaceForensics++ –DF	**82.0%**	**66.780%**
	FaceForensics++ –F2f	**69.67%**	
	Celeb-DF v2	**74.24%**	
	DFDC	**61.25%**	
Original [22]	FaceForensics++ –DF	75.27%	63.435%
	FaceForensics++ –F2f	67.37%	
	Celeb-DF v2	50.0%	
	DFDC	61.1%	

**Table 8:** Accuracy comparison of the VGG-16 model trained and tested on different datasets. The values in bold are the best performing for each dataset.

	Validation	FF++ –Deepfake	FF++ –Face2Face	DFDC	Celeb-DF	Overall
Trained						
FF++ –Deepfake		**AUROC: 0.878556**	AUROC: 0.710618	AUROC: 0.521114	AUROC: 0.528509	**0.6276**
		**Acc: 0.81995**	Acc: 0.6478	Acc: 0.5184	Acc: 0.5241	
FF++ –Face2Face		AUROC: 0.766970	**AUROC: 0.764427**	AUROC: 0.480113	AUROC: 0.531422	0.6001
		Acc: 0.6913	**Acc: 0.69675**	Acc: 0.4859	Acc: 0.52645	
DFDC		AUROC: 0.519190	AUROC: 0.476737	**AUROC: 0.650156**	AUROC: 0.476790	0.5226
		Acc: 0.5142	Acc: 0.48485	**Acc: 0.61225**	Acc: 0.4792	
CelebDF		AUROC: 0.529061	AUROC: 0.529152	AUROC: 0.464086	**AUROC: 0.806833**	0.5650
		Acc: 0.525	Acc: 0.5185	Acc: 0.4742	**Acc: 0.74245**	

**Table 9:** Different CNN models trained on TPU compared with the best-performing GPU model.

Model	Time Per Epoch	Total Time	Accuracy
VGG-16-GPU	440 s	183 min	82%
VGG-16	52 s	22 min	71.34%
VGG-19	57 s	24.5 min	63.56%
InceptionV3	72 s	30.2 min	58.72%
Xception	70 s	30 min	52.10%
ResNet50V2	55 s	23.1 min	68.37%
ResNet101V2	85 s	35.7 min	69.27%
ResNet152V2	110 s	46.3 min	70.50%

**Table 10:** Test results for all augmentations (1–4).

Augmentation	Training Time	Accuracy
No Augmentations	**183 min**	**82.0%**
Augmentation 1	672 min	77.5%
Augmentation 2	612 min	61.5%
Augmentation 3	212 min	76.0%
Augmentation 4	204 min	75.45%

## Data Availability

Datasets are available as explained in Section 3.2.
